# KSRP-PMR1-exosome association determines parathyroid hormone mRNA levels and stability in transfected cells

**DOI:** 10.1186/1471-2121-10-70

**Published:** 2009-09-23

**Authors:** Morris Nechama, Yong Peng, Osnat Bell, Paola Briata, Roberto Gherzi, Daniel R Schoenberg, Tally Naveh-Many

**Affiliations:** 1 Minerva Center for Calcium and Bone Metabolism, Nephrology Services, Hadassah-Hebrew University Medical Center, Jerusalem, Israel; 2 Department of Neurology, Columbia University College of Physicians and Surgeons, New York, NY, USA; 3 Istituto Nazionale per la Ricerca sul Cancro, Genova, Italy; 4 Department of Molecular & Cellular Biochemistry, The Ohio State University, Columbus, Ohio, USA

## Abstract

**Background:**

Parathyroid hormone (PTH) gene expression is regulated post-transcriptionally through the binding of the *trans-*acting proteins AU rich binding factor 1 (AUF1), Upstream of N-*ras *(Unr) and KH-type splicing regulatory protein (KSRP) to an AU rich element (ARE) in PTH mRNA 3'-UTR. AUF1 and Unr stabilize PTH mRNA while KSRP, recruiting the exoribonucleolytic complex exosome, promotes PTH mRNA decay.

**Results:**

PTH mRNA is cleaved by the endoribonuclease polysomal ribonuclease 1 (PMR1) in an ARE-dependent manner. Moreover, PMR1 co-immunoprecipitates with PTH mRNA, the exosome and KSRP. Knock-down of either exosome components or KSRP by siRNAs prevents PMR1-mediated cleavage of PTH mRNA.

**Conclusion:**

PTH mRNA is a target for the endonuclease PMR1. The PMR1 mediated decrease in PTH mRNA levels involves the PTH mRNA 3'-UTR ARE, KSRP and the exosome. This represents an unanticipated mechanism by which the decay of an ARE-containing mRNA is facilitated by KSRP and is dependent on both the exosome and an endoribonuclease.

## Background

Parathyroid hormone (PTH) regulates serum calcium and phosphate levels and bone strength. Serum calcium and phosphate concentrations, in turn, control PTH gene expression post-transcriptionally through regulated binding of the *trans-*acting proteins AU rich binding factor 1 (AUF1), Upstream of N-*ras *(Unr) and KH-type splicing regulatory protein (KSRP) to a type III AU rich element (ARE) in PTH mRNA 3'-UTR [1-3]. AUF1 and Unr stabilize PTH mRNA both in an in-vitro degradation assays (IVDA), using parathyroid extracts, and in intact cells. We have recently shown that the mRNA decay promoting protein KSRP decreases PTH mRNA stability and steady-state levels through the PTH mRNA ARE [3]. Both KSRP and AUF1 bind to PTH mRNA in vitro and in intact parathyroid glands [3]. In the parathyroid, the interaction of PTH mRNA with KSRP and AUF1 is regulated by changes in serum calcium and phosphate. Calcium depletion increases the association of AUF1 with the PTH mRNA ARE and decreases KSRP binding to the ARE resulting in mRNA stabilization. These interactions are reversed by phosphate depletion where PTH mRNA is destabilized [3]. In tranfected cells, over-expression of KSRP destabilizes the PTH mRNA and this is mediated by the PTH mRNA ARE. KSRP-PTH mRNA interaction is prevented by over-expression of AUF1 p45 isoform. Over-expression of AUF1 p45 also attenuates the KSRP-mediated destabilization of PTH mRNA in transfected cells [3]. The peptidyl-prolyl isomerase Pin1 is also a PTH mRNA destabilizing protein. Pin1 mediates its effects via interaction with KSRP, which leads to KSRP dephosphorylation and activation [4]. The regulated interactions of KSRP and AUF1 with the PTH mRNA ARE determine its half life in vivo and in vitro.

AREs are destabilizing elements located in the 3'UTRs of many inherently labile mRNAs [5]. AREs are targets for trans-acting proteins regulating mRNA localization, stability and translation [5]. Upon deadenylation, ARE-containing mRNAs are degraded in either a 3' to 5' or a 5' to 3' direction by two distinct exoribonucleolytic pathways mediated by the exosome and XrnI, respectively [6]. It has been recently demonstrated that these two pathways are functionally linked [7,8]. KSRP recruits the multiprotein 3'-5'exoribonuclease complex, exosome to target mRNAs. The central part of KSRP contains four adjacent KH domains that are required for its interaction with the decay-promoting machinery and with ARE containing mRNAs [9]. AU rich binding factor 1 (AUF1) promotes either decay or stabilization, depending on the mRNA and cell type [10].

In addition, a number of mRNAs are targeted by endonucleases that initiate decay by cleaving within the body of the mRNA while it is actively engaged by translating ribosomes. Three mRNA endonucleases have been linked to specific decay pathways; polysomal ribonuclease 1 (PMR1) [11], G3BP [12] and IRE-1 [13]. In Xenopus Laevis hepatocytes, many mRNAs are destabilized by estrogen through the activation of PMR1 [14]. PMR1 forms a selective complex with its substrate mRNA to initiate decay by cleaving within the mRNA [15]. Xenopus (x) PMR1 is a member of the peroxidase gene family and is synthesized as a 80-kDa precursor (PMR80) that is processed to the active 60-kDa form (PMR60) [11]. The ability of PMR1 to target polysomes and activate mRNA decay depends on tyrosine phosphorylation at position 650 in the C-terminus of PMR60 by c-Src [16,17].

Here we show that PTH mRNA is a substrate for PMR1 in vitro and in transfected cells. The PTH mRNA 3'-UTR ARE is required for PMR60-dependent PTH mRNA destabilization. PMR60 co-immunoprecipitates with PTH mRNA, the exosome and KSRP. Surprisingly, siRNA mediated knock-down of either exosome components or KSRP reduces the PMR1-mediated PTH mRNA decay in intact cells. We suggest that KSRP recruits a degradation complex, comprising both endo- and exo-ribonucleases to PTH mRNA, thus controlling its mRNA half-life.

## Results

### PMR60 decreases PTH mRNA and protein levels in transfected HEK293 cells by promoting ARE-dependent PTH mRNA decay

**Figure 1:**
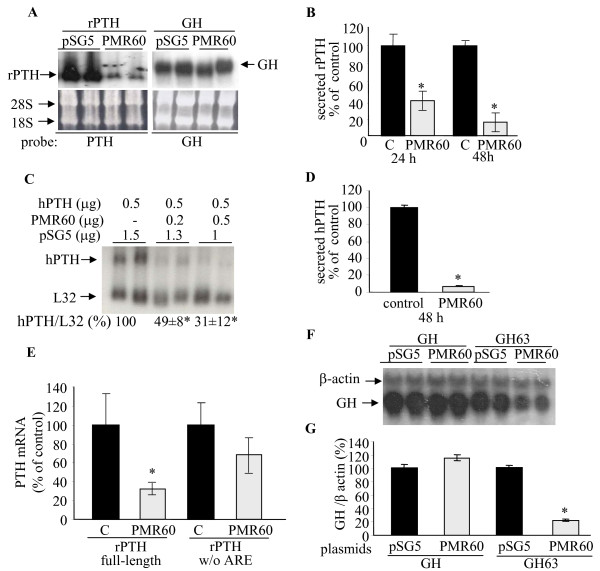
**PMR60 over-expression decreases PTH mRNA and protein levels in transfected HEK293 cells**. **A**. Northern blot analysis for rat (r) PTH or GH mRNA in HEK293 cells transiently co-transfected with plasmids for rat rPTH or GH and either catalytically active Myc-PMR60, or control (pSG5) plasmid at 48 h. Bottom panels: ethidium bromide staining of the membranes. Similar results were obtained in 4 independent experiments. **B**. Secreted rPTH in the medium of HEK293 cells in 3 repeat experiments as in A at 24 and 48 h. *, p < 0.05. **C**. Northern blot analysis for human (h) PTH mRNA in HEK293 cells transiently co-transfected with expression plasmids for hPTH and either PMR60 or a control plasmid (pSG5) at the indicated concentrations at 48 h. Quantification of PTH/L32 mRNA levels is shown below the gels. Similar results were obtained in 3 independent experiments. **D**. Secreted hPTH in the medium of HEK293 cells in 2 repeat experiments as in C. **E**. qRT PCR analysis for PTH and β-actin mRNA levels in HEK293 cells (n = 5) transfected with expression plasmids for rPTH or rPTH lacking the ARE and either catalytically active Myc-PMR60, or a control (pSG5) plasmid at 48 h. **F-G**. Effect of PMR60 over-expression on GH mRNA containing the PTH mRNA 63 nt ARE (GH63). **F**. Northern blot analysis of GH and control β-actin mRNA levels in cells transfected with plasmids for either GH or GH63 and PMR60 or control plasmids. **G**. Quantification of GH mRNA levels obtained in E and in an additional independent experiment, performed in triplicate. Results are shown as mean ± SE of mRNA in cells transfected with either GH or GH63 and pSG5 plasmid. *, p < 0.05.

Using an antibody to Xenopus PMR1 we identified the mammalian PMR1 ortholog in rat parathyroid, rat liver, and human HEK293 cells (unpublished observation). There is no parathyroid cell line and we therefore studied the effect of the catalytically-active form of TAP and Myc-tagged PMR1, PMR60 [18] on PTH mRNA levels in transfected cells. HEK293 cells were transiently co-transfected with plasmids expressing the endoribonuclease together with either rat (r) or human (h) PTH mRNA driven by a CMV promoter. PMR60 reduced rat (r) and human (h) PTH mRNA levels (Fig. 1A, left panel and C). PMR60 over-expression had no affect on the mRNA levels of transfected human growth hormone (GH) (Fig. 1A, right panel), luciferase (not shown), or endogenous L32 ribosomal protein mRNA (Fig. 1C). The decrease in PTH mRNA levels was matched by a parallel decrease in the amount of rPTH and hPTH produced and secreted into the growth medium of the cells expressing PMR60 (Fig. 1B and 1D).

PTH gene expression is largely controlled by an ARE in the 3'- UTR of PTH mRNA [3,19]. To determine if the regulation of PTH mRNA levels by PMR60 is exerted via the PTH mRNA ARE, We used a rPTH expression plasmid with an internal deletion of the PTH mRNA 3'-UTR ARE. In contrast to the full-length PTH mRNA, co-tranfected PMR60 had no effect on the mutated PTH mRNA (Fig. 1E). Therefore, the decrease in PTH mRNA levels by PMR60 is dependent on an intact ARE. We then used a GH reporter gene containing the rat PTH 63 nt ARE (GH63) [20]. Transfected PMR60 reduced GH63 mRNA levels but had no effect on wild type GH or endogenous β-actin mRNA levels (Fig. 1F-G). These results demonstrate that PMR60 specifically reduces steady-state PTH mRNA levels through the PTH mRNA ARE which is both necessary and sufficient for this effect.

**Figure 2:**
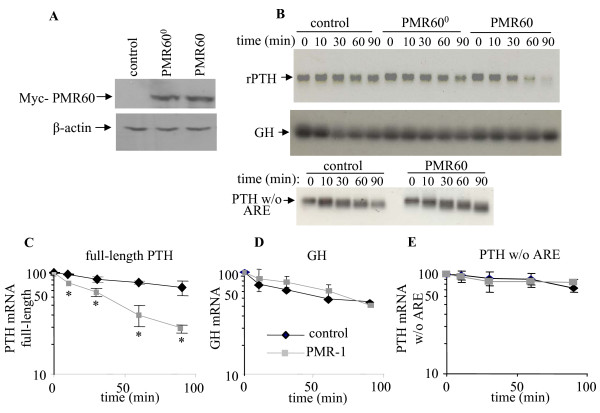
**PMR60 over-expression increases in vitro decay of PTH mRNA with an intact PTH mRNA 3'-UTR**. IVDA using radiolabeled polyadenylated full-length rat PTH mRNA (top panel), GH mRNA (middle panel) or polyadenylated rat PTH mRNA with an internal deletion of the PTH mRNA 3'-UTR ARE (bottom panel) and extracts from HEK293 cells transfected with either PMR60, the catalytically inactive form, PMR60^0^, or control pSG5 plasmid. **A**. Western blot analysis of protein extracts as above using anti Myc and β-actin antibodies, demonstrating equal amounts of Myc-PMR60 and Myc-PMR60^0 ^protein. **B**. RNA transcripts extracted at different time points, separated by agarose gels and visualized by autoradiography. **C-E**. Quantification of the IVDA results in A and in 2 repeat independent IVDA experiments, for PMR60 expressing or control extracts. Results are presented as mean ± S.E. of the amount of intact transcript at time 0 using a semi logarithmic scale. *, p < 0.05.

We then studied the effect of PMR60 on PTH mRNA decay by IVDA using uniformly radio-labeled polyadenylated full-length rat PTH mRNA, PTH mRNA lacking the ARE or GH mRNA. The transcripts were incubated with extracts from cells transfected with control plasmid, PMR60 or a catalytically inactive form of PMR60 (PMR60^0^) [18]. There were equal amounts of transfected PMR60 or PMR60^0 ^in the extracts (Fig. 2A). PMR60 over-expression accelerated PTH mRNA decay compared to control transfected cells or cells transfected with the catalytically-inactive form PMR60^0 ^(Fig. 2B, top panel and 2C). The t_1/2 _of the PTH mRNA transcript decreased from >100 min in the control cell extracts to ~50 min. in the PMR60 expressing cell extracts (Fig. 2C). Over-expression of PMR60 had no effect on the decay of a control GH mRNA (Fig. 2B, middle panel and 2D) or on PTH mRNA lacking the ARE (Fig. 2B bottom panel and E). Our results indicate that PMR60 over-expression specifically reduces PTH mRNA steady-state levels and stability acting through the PTH mRNA ARE.

### PMR60 associates with PTH mRNA in transfected cells and cleaves PTH mRNA in vitro

**Figure 3:**
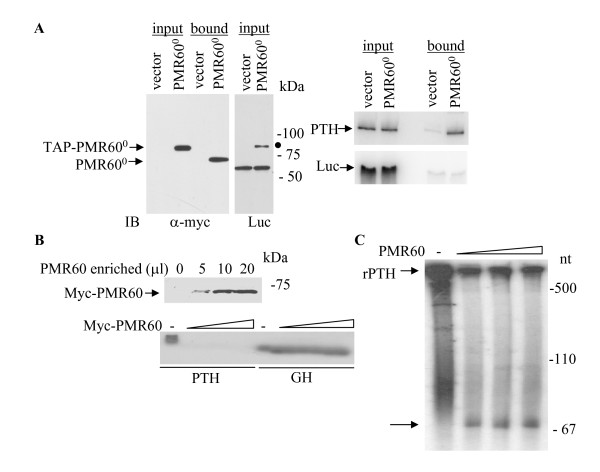
**PMR60 associates with PTH mRNA in transfected cells and cleaves PTH in vitro**. **A**. PMR60-PTH mRNA interaction. HEK293 cells were transiently co-transfected with TAP and Myc-tagged PMR60^0 ^(catalytically inactive) or with pcDNA3 (vector) and expression plasmids for hPTH and luciferase. PMR60^0 ^was affinity-purified followed by Tev protease cleavage. Left panels: Immunoblot (IB) analysis of the input (10% of the IPed fraction) and bound fractions using either anti-Myc antibody (left gel) or anti-luciferase antibody (right gel) to demonstrate transfection efficiency. A dot indicates nonspecific cross-reaction of anti-luciferase antibody with PMR60^0^. The decreased size of the recovered PMR60^0 ^(bound) results from Tev protease cleavage of the TAP tag. Right panels, RT-PCR analysis of RNA recovered from input and bound fractions, assayed for PTH (top gel) and luciferase (bottom gel) mRNAs. Similar results were obtained in 2 independent experiments. **B**. A PMR60 enriched fraction cleaves PTH mRNA in vitro. PMR60 was affinity-purified from cytoplasmic extracts of HEK293 cells transiently transfected with catalytically active PMR60 as in A. Top panel: IB analysis using anti-Myc antibody showing increasing concentrations of purified PMR60 used in the cleavage assay. Bottom panel: Uniformly radiolabeled rPTH or GH mRNAs without or with increasing amounts of PMR60 analyzed by agarose gel electrophoresis and autoradiography. **C**. 3'-end labeled rat PTH mRNA was treated with the amounts of PMR60 as in B. Cleavage products were analyzed by urea-PAGE and autoradiography to detect smaller intermediate products. The arrows mark the intact end-labeled PTH transcript and a single 3' cleavage product.

PMR60 forms a selective complex with its target mRNA and the substrate mRNA can be recovered with TAP-tagged catalytically-inactive PMR60^0 ^[18]. HEK293 cells were co-transfected with either TAP and Myc-tagged PMR60^0 ^or empty vector together with expression plasmids for either hPTH or luciferase. Immunoblot analysis using anti-Myc antibody identified PMR60^0 ^in the input and bound fractions (Fig. 3A, left panel). RT-PCR of RNA of the input and bound fractions showed that PTH mRNA was recovered by PMR60^0 ^and not by the negative control (Fig. 3A upper right panel). The specificity of this interaction was confirmed by the absence of luciferase mRNA in the PMR60^0^-recovered fraction (Fig. 3A bottom right panel). Therefore, PMR60^0 ^specifically interacts with PTH mRNA in HEK293 cells.

We then studied whether PTH mRNA is a substrate for partially purified PMR60 in vitro. We used the catalytically active form of TAP and Myc-tagged PMR1 (PMR60) [18], recovered by IgG-sepharose affinity purification from transfected cultured cells. PMR60 cleaved in vitro transcribed uniformly labeled PTH mRNA but not GH mRNA (Fig. 3B, bottom panel).

PMR60 also cleaved a 3'-end labeled rat PTH mRNA and generated a single 3'-end labeled cleavage product of approximately 70 nt, that was detected by urea-PAGE (Fig. 3C). A control GH transcript was not cleaved (data not shown), as with uniformly labeled GH mRNA (Fig. 3B). Our results suggest that PTH mRNA is a target for PMR60 in vitro and that this endoribonuclease cleaves PTH RNA at a site located approximately 70 nt from its 3' end.

### PMR60^0 ^associates with the exosome component Rrp4 and with KSRP

**Figure 4:**
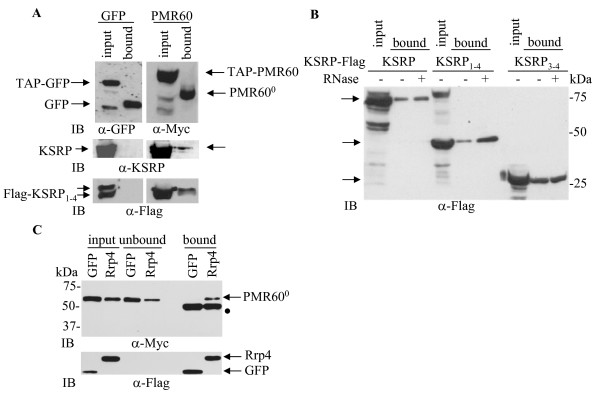
**PMR60 associates with KSRP and with the exosome component Rrp4**. **A-B**. KSRP-PMR60 interaction. **A**. HEK293 cells were transfected with plasmids for TAP and Myc-tagged PMR60 or TAP-tagged GFP without (middle panels, for endogenous KSRP) or with Flag-KSRP_1-4 _(bottom panels, for over-expressed Flag-KSRP_1-4_). TAP-PMR60 and TAP-GFP were affinity-purified followed by Tev protease cleavage and analyzed by immunoblots (IB) with the indicated antibodies, along side samples of the input (10% of the IPed fraction). The decreased size of the recovered PMR60 and GFP (top panel, bound) results from Tev protease cleavage of the TAP tags. **B**. HEK293 cells were co-transfected with expression plasmids for TAP-PMR60 as above and either Flag-KSRP or Flag-KSRP deletion mutants (Flag-KSRP_1-4 _and Flag-KSRP_3-4_). Cell extracts were divided into untreated (-) or RNase A and T1 treated (+) fractions. Input (10% of IPed fraction) and TAP-PMR60 purified fractions were analyzed by IB with anti-Flag antibodies. **C**. PMR60^0 ^co-immunoprecipitates with Rrp4. Cos-1 cells were co-transfected with plasmids expressing TAP- and Myc-PMR60^0 ^and either Flag-GFP or Flag-Rrp4. Cytoplasmic extracts were immunoprecipitated with anti-Flag agarose beads and analyzed by IB with the indicated antibodies. The dot in the upper blot indicates IgG heavy chain.

We have recently shown that KSRP interacts with the PTH mRNA ARE and decreases PTH mRNA stability and levels [3]. KSRP interacts with the exosome that is required for PTH mRNA rapid decay in the presence of parathyroid extracts [3]. We hypothesized that PMR1 may cooperate with KSRP and the exosome to facilitate PTH mRNA decay. We first studied the association of PMR60 with KSRP. HEK293 cells were co-transfected with expression plasmids for either TAP- and Myc-tagged PMR60 or GFP-TAP. GFP and PMR60 containing complexes were affinity purified, digested by Tev protease, and analyzed by immunoblot (Fig. 4A, upper panel). Anti-KSRP immunoblot demonstrated that endogenous KSRP co-purifies with PMR60 (Fig. 4A, middle panel). KSRP contains four adjacent KH domains [9]. KH domains 3-4 are sufficient for recruitment of the exosome complex and for KSRP association with ARE containing mRNAs including PTH mRNA [3,21]. We show that PMR60 also associated with transfected Flag-tagged wild type KSRP (Fig. 4B), KSRP_1-4 _containing all four KH domains without flanking sequences (Fig. 4A bottom panel and 4B), and with the deletion mutant Flag-KSRP_3-4 _(Fig. 4B) containing KH domains 3-4. These interactions were RNase insensitive (Fig. 4B). These data demonstrate that PMR60 associates with KSRP and that KH domains 3-4 are sufficient for this interaction.

We next studied the association of PMR60 with the exosome in Cos1 cells. PMR60 over-expression has a similar effect to decrease co-transfected PTH mRNA levels in these cells (not shown). Cos-1 cells were co-transfected with plasmids expressing TAP and Myc-tagged PMR60^0 ^and either Flag-GFP or Flag-Rrp4, one of the core exosome subunits. Cytoplasmic extracts were immunoprecipitated by anti-Flag antibody. Anti-Myc immunoblotting of the immuno-complexes revealed that PMR60^0 ^was specifically recovered by Rrp4 but not by GFP (Fig. 4C upper panel, bound). Altogether, our findings indicate that PMR60 associates with both KSRP and the exosome.

### KSRP, PMR60, and the exosome are components of the PTH mRNA decay machinery

**Figure 5:**
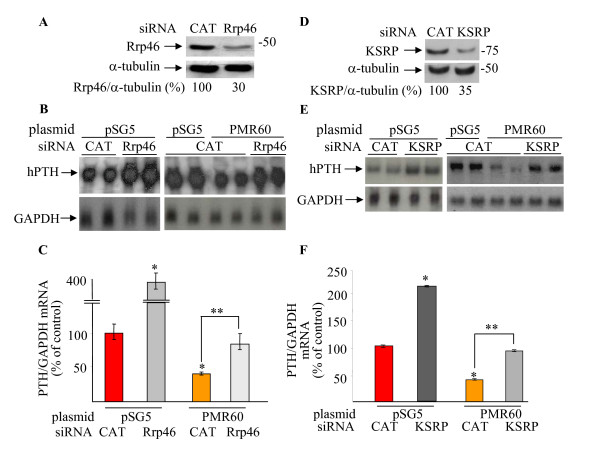
**The exosome and KSRP participate in the PMR60-induced decrease in PTH mRNA levels**. **A-C**. Rrp46 knock-down. HEK293 cells were transiently transfected in duplicate with either siRNA for Rrp46 or control CAT siRNA and expression plasmids for hPTH and either PMR60 or an empty vector (pSG5). **A**. Immunoblot analysis of HEK293 cell extracts 72 h after transfection. Rrp46 levels are presented below the gel as % of Rrp46 in extracts of cells transfected with CAT siRNA. **B**. Northern blot analysis of RNA extracted from cells transfected in duplicate as above. **C**. Quantification of the Northern blot data as in B and from 3 independent experiments. **D-F**. KSRP knock-down. HEK293 cells were transiently transfected in duplicate with either siRNA for KSRP or control CAT siRNA and expression plasmids for hPTH and either PMR60 or an empty vector (pSG5). **D**. Immunoblot analysis of HEK293 cell extracts 72 h after transfection and quantification of KSRP protein levels, presented below the gel. **E**. Northern blot analysis of RNA extracted from cells transfected in duplicate as above. **F**. quantification of Northern blot data from 3 repeat experiments including the one in E. Results in C and F are presented as mean ± SE of mRNA levels compared to cells transfected with CAT siRNA and pSG5 plasmid (*, p < 0.05) or compared to CAT siRNA and PMR60 transfected cells (**, p < 0.05).

To further study the mechanism of PTH mRNA decay by PMR1 and the exosome, PMR60 over expression was performed together with siRNA-mediated knock-down of exosome components. A ~70% Rrp46 knock-down compared to control (CAT) siRNA (Fig. 5A), led to a marked increase in the mRNA levels of co-transfected PTH (Fig. 5B and 5C), thus confirming our previous data that the exosome is required for PTH mRNA decay [3]. As expected, PMR60 reduced PTH mRNA levels when co-transfected with the control siRNAs (Fig. 5B and 5C, see also Fig. 1). Simultaneous over-expression of PMR60 and knock-down of Rrp46 lead to PTH mRNA levels of control transfected cells (Fig. 5B and 5C). Neither siRNAs affected mRNA levels of the endogenous control GAPDH mRNA (Fig. 5B, bottom panel). Similar results were obtained by knocking-down the exosome subunit Rrp40 (data not shown). These results indicate that exosome components participate in the PMR60-induced decrease in PTH mRNA in intact cells.

We also studied the ability of PMR1 to reduce PTH mRNA levels in KSRP depleted cells. KSRP was knocked down by specific siRNAs in cells expressing PTH and either PMR60 or control plasmid. KSRP knock-down (~60%, Fig. 5D) increased PTH mRNA levels (Fig. 5E and 5F) as we have previously reported [3]. Interestingly, KSRP knock-down partially prevented the PMR60-induced decrease in PTH mRNA levels (Fig. 5E and 5F). Similar results were obtained using an additional set of siRNAs targeting a different sequence of KSRP mRNA (data not shown). Altogether our results indicate that the exosome and KSRP participate in the PMR60-dependent decrease in PTH mRNA levels in cultured cells.

## Discussion

PTH mRNA contains a 63 nt-long ARE-like region in its 3'-UTR that determines PTH mRNA stability [20,22]. This element leads to decreased mRNA levels of reporter genes in transfected cells [20,23]. The regulated binding of the PTH mRNA stabilizing proteins, AUF1 and Unr, and the destabilizing factor KSRP to this 63 element controls PTH mRNA levels in vivo in the parathyroid gland and in vitro in transfected cells [1-3]. KSRP promotes rapid mRNA decay by recruiting the exoribonucleolytic complex exosome to its target mRNAs [21]. We now show that the endoribonuclease PMR1 decreases PTH mRNA levels in transfected cells and that this involves the PTH mRNA ARE, KSRP and the exosome.

Few vertebrate mRNA endonucleases have been identified, one of which is PMR1. Since a PMR1 immunoreactive protein similar to the mammalian ortholog [24] is expressed in the parathyroid, we asked whether this endoribonuclease may be part of the PTH mRNA decay machinery. Reagents for mammalian PMR1 are not available so we used the closely-related Xenopus protein [18]. Over-expression of the catalytically active form of PMR1 (PMR60) decreased PTH mRNA levels in co-transfected cells and this was dependent upon the PTH mRNA 63 nt ARE. Similarly, in IVDA experiments, extracts from PMR60 over-expressing cells led to a more rapid decay of PTH mRNA with an intact ARE, when compared to extracts from mock-transfected cells. These results identify PTH mRNA and in particular the PTH mRNA ARE as a target for PMR1 in transfected cells. A PMR60 enriched fraction also specifically cleaved PTH mRNA in vitro. At least one cleavage site was identified ~70 nt from the PTH mRNA 3' end. It has been reported that PMR1 preferentially cleaves single-stranded RNAs at UG dinucleotides within albumin mRNA [25]. The PTH mRNA 3'-UTR is an open region with little folded base pairing [20] and contains several UG dinucleotides that may be potential targets for PMR1. Specifically, the 3' terminal region of rat PTH mRNA contains a single UG dinucleotide compatible with the production of a ~70 nt 3' PTH mRNA fragment upon digestion. Interestingly, this UG dinucleotide is part of the PTH mRNA 3'-UTR 63 nt ARE instability element that is both necessary and sufficient to confer regulation of PTH mRNA stability by changes in calcium and phosphate levels by rat parathyroid extracts and in vitro in transfected cells [19,20,22].

We also show that PMR60^0 ^specifically interacts with PTH mRNA. Furthermore, PMR60 displays an unanticipated association with the exosome component Rrp4 and with KSRP. Both interactions occur in the absence of PTH mRNA. It is of interest that KSRP KH domains 3-4 that mediate KSRP-exosome association and promote ARE-containing mRNA decay [21], are sufficient for KSRP-PMR60 association. KH domains 3 and 4 also mediate the binding of KSRP to the PTH mRNA ARE [3]. We can hypothesize that KSRP recruits PMR1 to additional labile mRNAs and this protein-protein association would facilitate KSRP-dependent decay promoting activity.

**Figure 6:**
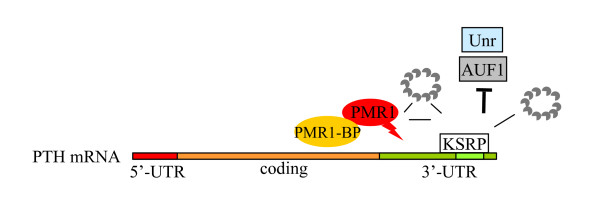
**Model for the role of PTH mRNA interacting proteins in PTH mRNA stability**. PTH mRNA stability is regulated through the interaction of AUF1, Unr and KSRP with the PTH mRNA 3'-UTR ARE (light green) [1-3]. KSRP, the exosome and PMR1 are recruited to PTH mRNA through KSRP-exosome interaction leading to decreased PTH mRNA stability and levels. PMR1-PTH mRNA association may be mediated by additional unidentified binding protein/s (PMR1-BP).

Our results show that the effect of the endoribonuclease PMR1 on PTH mRNA levels is dependent upon the expression of exosome components and KSRP, which interacts with both the exosome and PMR1. These results suggest that PMR1, KSRP and the exosome participate in PTH mRNA decay by forming a multi-subunit degradation complex with the PTH mRNA ARE (Fig. 6). Upon PMR1 cleavage, PTH mRNA decay may proceed through exosome mediated removal of the 5' fragment or of both fragments by exonucleolytic cleavage. Of interest, PMR60 over-expression prevented the increased PTH mRNA levels induced by either exosome or KSRP depletion, suggesting that PMR60 may also have an effect on PTH mRNA expression that is independent of both KSRP and the exosome.

Cooperation between ribonucleolytic machineries have been reported also in other systems. Knock-down of the de-capping protein Dcp2 reduces decay from the 3' end of a β-globin mRNA carrying the c-fos ARE, and knockdown of the exosome subunits PM/Scl-100 or Rrp41 reduce decay from the 5' end, indicating that the 5' and 3' decay pathways are functionally linked and that unstable mRNAs can be degraded simultaneously from both ends [7,8]. Wang and Kiledjian have similarly shown that mRNA decay proceeds through a coupled 3' to 5' and 5' to 3' exoribonucleolytic pathway that involves the interaction of a sub-set of the exosome proteins with DcpS and the decapping pathway [26]. Moreover, it was recently shown that eukaryotic exosome itself contains both exonuclease and endonuclease activity, mediated by two distinct domains of its Dis3 (Rrp44) subunit. [27,28]. Our studies do not exclude the possibility of an endonuclease activity of the exosome that co-purifies with PMR60 and is distinct from PMR60 and cleaves PTH mRNA. The combination of endo and exoribonucleases in one RNA-degrading machine may offer a fundamental advantage to the cell and appears to be more widespread in nature than could be expected [28]. Altogether, the above observations and the data presented here demonstrate that interactions of the exosome complex with other mRNA decay enzymes facilitate and coordinate mRNA decay, both endo- and exonucleolytically.

## Conclusion

PTH mRNA is a target for the endonuclease PMR1. The PMR1 mediated decrease in PTH mRNA levels involves the PTH mRNA 3'-UTR ARE, KSRP and the exosome. Our findings suggest an unanticipated mechanism by which KSRP and the exosome regulate the half-life of a target mRNA by facilitating its endoribonucleolytic cleavage.

## Methods

### Protein extractions

For IVDA post-mitochondrial extracts were prepared. Cultured cells were incubated on ice for 10 min in an extraction buffer containing 0.25 M sucrose, 30 mM Tris HCl pH 7.5, 2 mM DTT, and a protease inhibitor mix. Samples were homogenized and the supernatant cleared by centrifugation at 15,000 g for 15 min (4°C). For Western blots, extracts from cultured cells were prepared using RIPA buffer containing 150 mM NaCl, 1% NP40, 0.5% sodium deoxycholate, 0.1% SDS and protease inhibitors. Extracts were stored in aliquots at -80°C.

### Cell cultures and transfections

HEK293 cells were transiently transfected with different expression plasmids using a Ca-P transfection kit (Sigma, St Louis MO). siRNA oligonucleotides were transiently co-transfected with expression plasmids using Lipofectamine 2000 Reagent (Invitrogen, Carlsbad, CA).

### PMR60 purification and in vitro activity assay

Xenopus (x) PMR60 was purified as previously described [18]. Briefly, an expression plasmid for active form of xPMR1 (Myc-PMR60-TAP) was transfected into HEK293 cells and after 48 h PMR60 was recovered from cell lysate by IgG-Sepharose 6 Fast Flow (Amersham, Little Chalfont UK), followed by Tev protease cleavage of the TAP tag. Purified PMR60 was incubated with uniformly labeled PTH mRNA transcript for 1 h at room temperature in a reaction buffer containing 30 mM Tris HCl pH 7.5, 1 mM DTT, 2 mM MgCl_2 _and 75 mM KCl.

### PTH mRNA recovery by PMR60^0^

HEK293 cells were transiently co-transfected with the catalytically inactive PMR form, xPMR60^0^, and expression plasmids for either the hPTH gene or luciferase cDNA. xPMR60^0 ^containing complexes were recovered from cell extracts on IgG-Sepharose and cleavage with Tev protease. RNA was extracted using TRIzol reagent (Invitrogen, Carlsbad, CA) and reverse transcribed using Super-Script II Reverse Transcriptase. cDNA was analyzed by semi quantitative PCR with γ [^32^P] ATP.

### PMR60-exosome association

Cos-1 cells were co-transfected with constructs for xPMR60^0 ^and either Flag-GFP or Flag-Rrp4. Cytoplasmic extracts were applied to monoclonal anti-Flag M2 agarose beads (Sigma, St Louis MO), eluted and analyzed by SDS-PAGE and Western blots.

### PMR60-KSRP association

HEK293 cells were co-transfected with expression plasmids for either TAP and Myc-tagged xPMR60^0 ^or GFP-TAP, with or without expression plasmid for Flag-tagged KSRP or truncated KSRP containing the different KH domains. GFP and PMR60 were recovered by IgG-Sepharose 6 Fast Flow, digested by Tev protease, and analyzed by immunoblots for endogenous KSRP using an anti KSRP antibody, or for the transiently transfected Flag-tagged KSRP using anti Flag antibody. In some experiments 40 μg/ml of RNase A and 25 μg/ml of RNase T1 were added to the protein extracts to determine if interactions were RNA dependent. After incubated at room temperature for 20 min. proteins were recovered and analyzed by immunoblots as above.

### siRNAs

Previously published siRNAs targeting Rrp46 :5'-CAAGGCCACACUCGAAGUG-3', Rrp40: 5'-GGAGACCAUGUGAUUGGCA-3' [9], KSRP: 5'-AAGATCAACCGGAGAGCAAGA-3' [29] and an additional set of commercial, siRNAs for KSRP (sequence not available) and the control CAT: 5' r(GACGGUGAGCUGGUGAUAU)d(TT)-3' or TTCTCCGAACGAACGTGTCACGT-3', were all synthesized by QIAGEN (Hilden, Germany).

### Northern blots

RNA was extracted with Tri-Reagent (Molecular Research Center, Cincinatti, OH) and analyzed as previously described [20].

### qRT-PCR

RNA was reverse transcribed with random hexamer primers using a Maxime RT premix kit (iNtRON Biotechnology, Gyeonggi-do, Korea), and analyzed by real-time quantitative polymerase chain reaction (qPCR) using ABI Prism 7901 Sequence Detection System (Applied Biosystems, Foster City, CA, USA) and SYBR Green ROX Mix (ABgene, Epsom, UK).

### PCR primers for qRT-PCR

Rat PTH primers were: 5'-TTGTCTCCTTACCCAGGCAGAT-3' and 5'-TTTGCCCAGGTTGTGCATAA-3'. Primers for β-actin were: 5'-CAGGCATTGCTGACAGGATG-3' and 5'-CTCAGGAGGAGCAATGATCTTGAT-3'.

### Immunoblots

Proteins were analyzed by SDS PAGE immunoblots as previously described [30].

### RNA transcription and labeling

Uniformly α[^32^P] UTP labeled polyadenylated RNAs for the full-length PTH mRNA, a PTH mRNA with an internal deletion of the ARE or GH mRNA were transcribed *in vitro *as previously described [3,19]. For 3'-end labeled PTH mRNA transcripts, the rat PTH cDNA containing plasmid was linearized with BclI and unlabeled RNA transcribed and extracted using Tri Reagent. RNA was then annealed to a primer homologous to the 3'-end of the transcript, leaving a 2 base 5' overhang. The sequence of the primer was 5'-TGATTAAACTTT-3'. The 3'-end of the transcript was then labeled using 4 U of Klenow enzyme, 2 μM dNTP and α[^32^P] dCTP by incubation at 37°C for 2 h. All labeled transcripts were purified using mini Quick Spin RNA Columns (Roche, Mannheim, Germany).

### In Vitro Degradation Assays (IVDA)

Radiolabeled transcripts were incubated with 50 μg proteins of post mitochondrial cell extracts in a reaction buffer containing 3 mM Tris HCl, pH 7.5, 2 mM MgCl_2_, 3 mM NaCl, 10 mM ATP and 80 units/ml RNasin and analyzed as described [3]. At timed intervals samples were removed separated on formaldehyde agarose gels or urea SDS PAGE and analyzed by autoradiography.

### Plasmids

Rat PTH cDNA was cloned in either pcDNA3 [2] for transient transfections or in pBluescript II KS [22] for in vitro transcription for IVDAs. The pBluescript II KS plasmid containing the full-length rat PTH cDNA including a stretch of ~150 dT nucleotides that by in vitro transcription produced a poly A tail was used. An internal BsaI-BclI fragment (~80 bp) was removed by partial restriction enzyme digestion of the pBluescript II KS-PTH cDNA plasmid, followed by ligation to produce a plasmid without the ARE [3]. The human PTH gene was in pcDNA3 [3]. The firefly luciferase plasmid was in pGL3 (Promega, Madison WI). The GH gene expression plasmid was kindly provided by O. Meyuhas, Hadassah Medical School, Jerusalem, Israel) [31]. The GH-PTH mRNA 63 nt plasmid (GH63) was previously described [20] and contained the 63 nt rat PTH mRNA ARE cloned between the 3' end of the GH mRNA coding sequence and the GH mRNA 3'-UTR. The corresponding GH cDNAs cloned into pBluescript II KS were used for in vitro transcription. Plasmids coding for the active (PMR60) or inactive (PMR60^0^) forms of Xenopus PMR1 contained a N terminal Myc tag and a C terminal TAP tag and were in pcDNA3 [32]. KSRP in pcDNA3 contained either Flag-tagged full-length KSRP or different KSRP KH domains [21]. Empty control vectors (pcDNA3 and pSG5) were used as indicated.

### Antibodies

Anti-KSRP was previously described [21]. The anti-Flag, anti α-tubulin, and anti-GFP were from Sigma (St Louis MO). Anti-Myc was from Cell Signaling (Boston, MA, USA)

### Serum PTH measurements

Serum rat/human PTH was measured using the rat/human intact PTH ELISA Kits (Immunotopics, San Clemente, California, USA).

### Statistical analysis

Values are reported as mean ± SEM unless stated otherwise. A 2-tailed *p *value was considered significant when less than 0.05.

## Abbreviations

ARE: AU rich element; AUF1: AU rich binding factor 1; GH: growth hormone; HEK: human embryonic kidney; IVDA: in vitro degradation assay; KSRP: KH-type splicing regulatory protein; PMR: polysomal ribonuclease; PTH: parathyroid hormone; qRT PCR: quantitative reverse trasnscriptase polymerase chain reaction; siRNA: small interfering RNAs; Unr: Upstream of N-*ras*; UTR: untranslated region.

## Authors' contributions

MN designed, performed the experiments, analyzed and interpreted data and drafted the manuscript; YP and DS designed and YP performed the PMR1-exosome-PTH mRNA interactions, TN-M conceived the study, interpreted data and wrote the manuscript; RG, PB and DS interpreted data and critically reviewed the manuscript. All authors read and approved the final manuscript.

## References

[B1] Sela-Brown A, Silver J, Brewer G, Naveh-Many T (2000). Identification of AUF1 as a parathyroid hormone mRNA 3'-untranslated region binding protein that determines parathyroid hormone mRNA stability. J Biol Chem.

[B2] Dinur M, Kilav R, Sela-Brown A, Jacquemin-Sablon H, Naveh-Many T (2006). In vitro evidence that upstream of N-ras participates in the regulation of parathyroid hormone messenger ribonucleic acid stability. Mol Endocrinol.

[B3] Nechama M, Ben Dov IZ, Briata P, Gherzi R, Naveh-Many T (2008). The mRNA decay promoting factor K-homology splicing regulator protein post-transcriptionally determines parathyroid hormone mRNA levels. FASEB J.

[B4] Nechama M, Uchida T, Yosef-Levi IM, Silver J, Naveh-Many T (2009). The peptidyl-prolyl isomerase Pin1 determines parathyroid hormone mRNA levels and stability in rat models of secondary hyperparathyroidism. J Clin Invest.

[B5] Barreau C, Paillard L, Osborne HB (2006). AU-rich elements and associated factors: are there unifying principles?. Nucl Acids Res.

[B6] Garneau NL, Wilusz J, Wilusz CJ (2007). The highways and byways of mRNA decay. Nat Rev Mol Cell Biol.

[B7] Murray EL, Schoenberg DR (2007). A+U-rich instability elements differentially activate 5'-3' and 3'-5' mRNA decay. Mol Cell Biol.

[B8] Stoecklin G, Mayo T, Anderson P (2006). ARE-mRNA degradation requires the 5'-3' decay pathway. EMBO Rep.

[B9] Chou CF, Mulky A, Maitra S, Lin WJ, Gherzi R, Kappes J, Chen CY (2006). Tethering KSRP, a decay-promoting AU-rich element-binding protein, to mRNAs elicits mRNA decay. Mol Cell Biol.

[B10] Wilusz CJ, Wilusz J (2004). Bringing the role of mRNA decay in the control of gene expression into focus. Trends Genet.

[B11] Chernokalskaya E, Dubell AN, Cunningham KS, Hanson MN, Dompenciel RE, Schoenberg DR (1998). A polysomal ribonuclease involved in the destabilization of albumin mRNA is a novel member of the peroxidase gene family. RNA.

[B12] Gallouzi IE, Parker F, Chebli K, Maurier F, Labourier E, Barlat I, Capony JP, Tocque B, Tazi J (1998). A novel phosphorylation-dependent RNase activity of GAP-SH3 binding protein: a potential link between signal transduction and RNA stability. Mol Cell Biol.

[B13] Hollien J, Weissman JS (2006). Decay of endoplasmic reticulum-localized mRNAs during the unfolded protein response. Science.

[B14] Cunningham KS, Dodson RE, Nagel MA, Shapiro DJ, Schoenberg DR (2000). Vigilin binding selectively inhibits cleavage of the vitellogenin mRNA 3'-untranslated region by the mRNA endonuclease polysomal ribonuclease 1. Proc Natl Acad Sci USA.

[B15] Cunningham KS, Hanson MN, Schoenberg DR (2001). Polysomal ribonuclease 1 exists in a latent form on polysomes prior to estrogen activation of mRNA decay. Nucleic Acids Res.

[B16] Peng Y, Schoenberg DR (2007). c-Src activates endonuclease-mediated mRNA decay. Mol Cell.

[B17] Peng Y, Liu X, Schoenberg DR (2008). The 90-kDa heat shock protein stabilizes the polysomal ribonuclease 1 mRNA endonuclease to degradation by the 26S proteasome. Mol Biol Cell.

[B18] Yang F, Peng Y, Schoenberg DR (2004). Endonuclease-mediated mRNA decay requires tyrosine phosphorylation of polysomal ribonuclease 1 (PMR1) for the targeting and degradation of polyribosome-bound substrate mRNA. J Biol Chem.

[B19] Kilav R, Silver J, Naveh-Many T (2001). A conserved cis-acting element in the parathyroid hormone 3'-untranslated region is sufficient for regulation of RNA stability by calcium and phosphate. J Biol Chem.

[B20] Kilav R, Bell O, Le SY, Silver J, Naveh-Many T (2004). The parathyroid hormone mRNA 3'-untranslated region AU-rich element is an unstructured functional element. J Biol Chem.

[B21] Gherzi R, Lee KY, Briata P, Wegmuller D, Moroni C, Karin M, Chen CY (2004). A KH domain RNA binding protein, KSRP, promotes ARE-directed mRNA turnover by recruiting the degradation machinery. Mol Cell.

[B22] Moallem E, Silver J, Kilav R, Naveh-Many T (1998). RNA protein binding and post-transcriptional regulation of PTH gene expression by calcium and phosphate. J Biol Chem.

[B23] Bell O, Silver J, Naveh-Many T (2005). Identification and characterization of *cis*-acting elements in the human and bovine parathyroid hormone mRNA 3'-untranslated region. J Bone & Min Res.

[B24] Bremer KA, Stevens A, Schoenberg DR (2003). An endonuclease activity similar to Xenopus PMR1 catalyzes the degradation of normal and nonsense-containing human beta-globin mRNA in erythroid cells. RNA.

[B25] Chernokalskaya E, Dompenciel R, Schoenberg DR (1997). Cleavage properties of an estrogen-regulated polysomal ribonuclease involved in the destabilization of albumin mRNA. Nucleic Acids Res.

[B26] Wang Z, Kiledjian M (2001). Functional link between the mammalian exosome and mRNA decapping. Cell.

[B27] Lebreton A, Tomecki R, Dziembowski A, Seraphin B (2008). Endonucleolytic RNA cleavage by a eukaryotic exosome. Nature.

[B28] Schaeffer D, Tsanova B, Barbas A, Reis FP, Dastidar EG, Sanchez-Rotunno M, Arraiano CM, van Hoof A (2009). The exosome contains domains with specific endoribonuclease, exoribonuclease and cytoplasmic mRNA decay activities. Nat Struct Mol Biol.

[B29] Pullmann R, Kim HH, Abdelmohsen K, Lal A, Martindale JL, Yang X, Gorospe M (2007). Analysis of Turnover and Translation Regulatory RBP Expression Through Binding to Cognate mRNAs. Mol Cell Biol.

[B30] Bell O, Gaberman E, Kilav R, Levi R, Cox KB, Molkentin JD, Silver J, Naveh-Many T (2005). The protein phosphatase calcineurin determines basal parathyroid hormone gene expression. Molecular Endocrinology.

[B31] Levy S, Avni D, Hariharan N, Perry RP, Meyuhas O (1991). Oligopyrimidine tract at the 5' end of mammalian ribosomal protein mRNAs is required for their translational control. Proc Natl Acad Sci USA.

[B32] Yang F, Schoenberg DR (2004). Endonuclease-mediated mRNA decay involves the selective targeting of PMR1 to polyribosome-bound substrate mRNA. Mol Cell.

